# Malignant fibrous histiocytoma of the distal femur after an arthroscopic anterior cruciate ligament reconstruction: A case report and a review of the literature

**DOI:** 10.1186/1471-2407-10-264

**Published:** 2010-06-08

**Authors:** Turgay Efe, Thomas J Heyse, Markus D Schofer, Susanne Fuchs-Winkelmann, Peter Rexin, Jan Schmitt

**Affiliations:** 1 Department of Orthopaedics and Rheumatology, University Hospital Marburg, Baldingerstrasse, 35043 Marburg, Germany; 2 Institute of Pathology, University Hospital Marburg, Baldingerstrasse, 35043 Marburg, Germany

## Abstract

**Background:**

Malignant degeneration in association with orthopaedic implants is a known but rare complication. To our knowledge, no case of osseous malignant fibrous histiocytoma after anterior cruciate ligament reconstruction is reported in the literature.

**Case presentation:**

**We report a **29-year-old male Turkish patient who presented with severe pain in the operated knee joint 40 months after arthroscopic anterior cruciate ligament reconstruction. X-ray and MR imaging showed a large destructive tumor **in **the medial femoral condyle. Biopsy determined a malignant fibrous histiocytoma. After neoadjuvant chemotherapy, wide tumor resection and distal femur reconstruction with a silver-coated non-cemented tumor knee joint prosthesis was performed. Adjuvant chemotherapy was continued according to the EURAMOS 1 protocol.

**Conclusions:**

Though secondary malignant degeneration after orthopaedic implants or prostheses is not very likely, the attending physician should take this into consideration, especially if symptoms worsen severely over a short period of time.

## Background

The continuous increase in recreational sports leads to a continuously increasing number of capsule and ligament injuries of the knee joint as well. About 20% of the knee injuries are accompanied by anterior cruciate ligament (ACL) ruptures [1]. Reconstruction of the ACL belongs to the therapies of choice for the sportively active patient and is one of the most common surgical interventions for knee ligament reconstruction [2].

The primary malignant fibrous histiocytoma (MFH) has been described for the first time as a soft tissue tumor in 1964 by O'Brien and Stout [3]. MFH occurs only rarely as primary bone tumor and appears mainly on the meta- and diaphysis of the long bones [4]. MFH usually occurs in older age with a peak in the 6^th ^decade of life; men are affected more often than women [5]. The development of secondary osseous malignant fibrous histiocytoma in association with orthopaedic implants [6] or prostheses [7] is a rare but nevertheless **a **known complication. Secondary malignant fibrous histiocytoma has been desribed at sites of pre-existing bone lesions [8], after irradiation treatment [9] and burn injuries [10].

In the case history presented below, we report the manifestation of an osseous MFH at the distal femur 40 months after arthroscopic anterior cruciate ligament reconstruction using autologous semitendinosus tendon. To our knowledge, no case of an osseous MFH after anterior cruciate ligament reconstruction is reported in the literature.

## Case presentation

**Figure 1:**
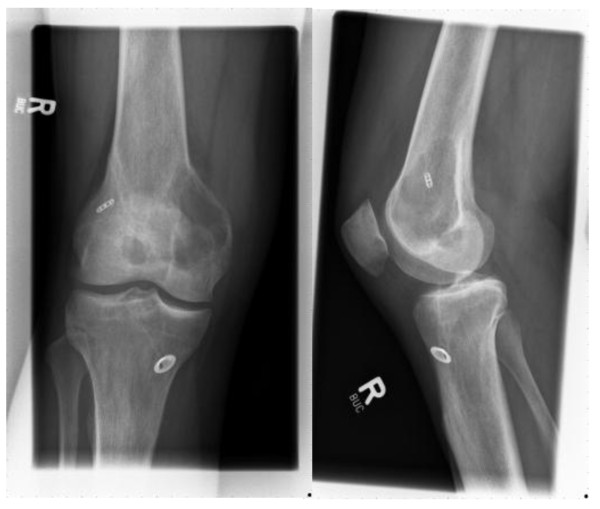
**Conventional X-ray imaging in anteroposterior (a) and lateral (b) projection show a 5.6 × 5 cm osteolysis of the medial femoral condyle with infiltration into surrounding soft tissue after arthroscopic anterior cruciate ligament reconstruction using the semitendinosus tendon**.

In January 2006, a 26-year-old male had sustained an isolated rupture of the right cruciate ligament during a soccer game. This was diagnosed by a stability investigation with a positive Lachman and Pivot shift test and was confirmed by MRI. The knee joint was without signs of irritation. ROM was 0°-140°. Ten days later, arthroscopic ACL reconstruction was performed using a quadruple autologous ipsilateral semitendinosus tendon graft. The fixation elements used were a titanium Endobutton (Smith & Nephew, Schenefeld, Germany) on the femoral side and a titanium Suture Disc (B. Braun, Aesculap AG, Tuttlingen, Germany) on the tibial side, respectively. The peri- and postsurgical course was free of complications. 10 months after ligament reconstruction, the patient could resume sports activity and was very satisfied with the operative outcome. 40 months after surgery, the patient presented with strongly increasing pain in the medial femoral condyle of the operated knee joint. Clinical investigation showed a stable knee without signs of soft tissue irritation. Non-contrast radiology showed a space-occupying lesion of 5.6 × 5 cm at the medial femoral condyle, mainly located in the metaphysis (Figure 1a and 1b). The margins showed partly a sclerotic zone and partly an infiltrating margin. There was no obvious soft tissue shadow.

**Figure 2:**
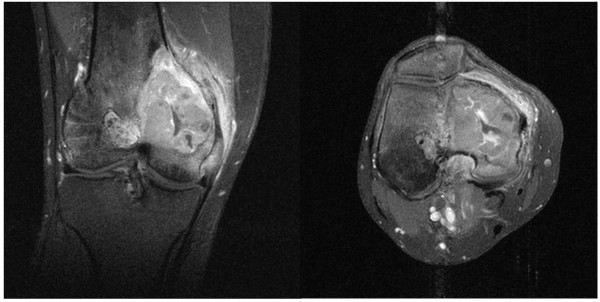
**Coronary (a) and transversally (b) T1-weighted MRI sequence shows a bone tumor spreading across the medial cortical bone and infiltrating into the vastus medialis muscle and the medial retinaculum**. The graft and the femoral drill tunnel are not infiltrated by the MFH.

**Figure 3:**
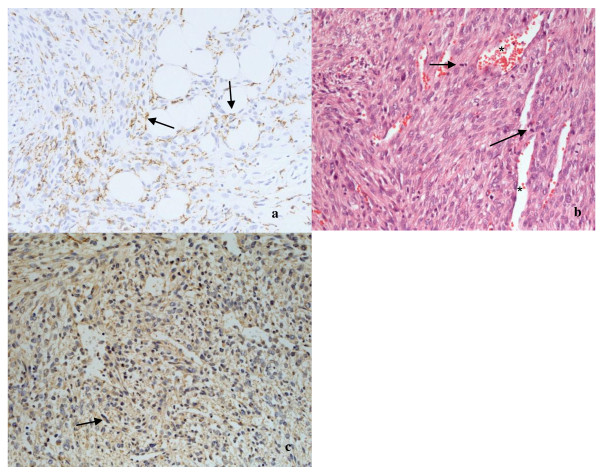
(a) shows the tumor biopsy with CD68 positive histiocytes (black arrows, 200-fold magnification), (b) shows the histological picture of a cell-rich mesenchymal tumor with storiform growth pattern, marked polymorphism, high rate of mitosis (black arrows) and marked angioneogenesis (*); HE, 200-fold magnification. (c) demonstrates the **clear sm actin immunoreactivity **(black arrow) of the tumor cells; anti-sm actin, 100-fold magnification.

MRI with contrast agent (Gadolinium) revealed a bone tumor of 6.2 × 5 × 4.2 cm. The bone tumor had infiltrated the medial retinaculum and the vastus medialis muscle (Figure 2a and 2b). Computertomographic staging investigation did not show any intrathorakal and intra-abdominal metastases. Three-phase skeletal scintigraphy with intravenous injection of 669.5 MBq Tc-99 m HDP did not show any further pathological lesions, except from the lesion at the right medial femoral condyle with increased accumulation in the early and late phase. For diagnosis of the tumor entity, an open biopsy of the space-occupying lesion was performed. The tumor was a highly cellular malignant mesenchymal tumor. It was composed of spindle cells mainly. Areas of necrosis, increased vascularity and CD68 positive histocytes (Figure 3a) were seen. Due to its storiform growth pattern, moderate nuclear pleomorphy, strongly enhanced proliferative activity and immunohistochemical marker profile, it was classified as high-grade fibrous histiocytoma (Figure 3b and 3c). The strong positive immune reaction against smooth muscle actin antibodies did not support the possibility of a fibrosarcoma of the bone. Differential diagnosis of a leiomyosarcoma of the bone had to be taken into account, but this was less likely because the tumor was negative for desmin and myogenin. After presenting the patient in the interdisciplinary tumor conference, he was included into the osteosarcoma register (Prof. Bielack, Olgahospital Stuttgart, Germany). 2 chemotherapy cycles were performed over a 10 week period according to the EURAMOS 1 protocol.

**Figure 4:**
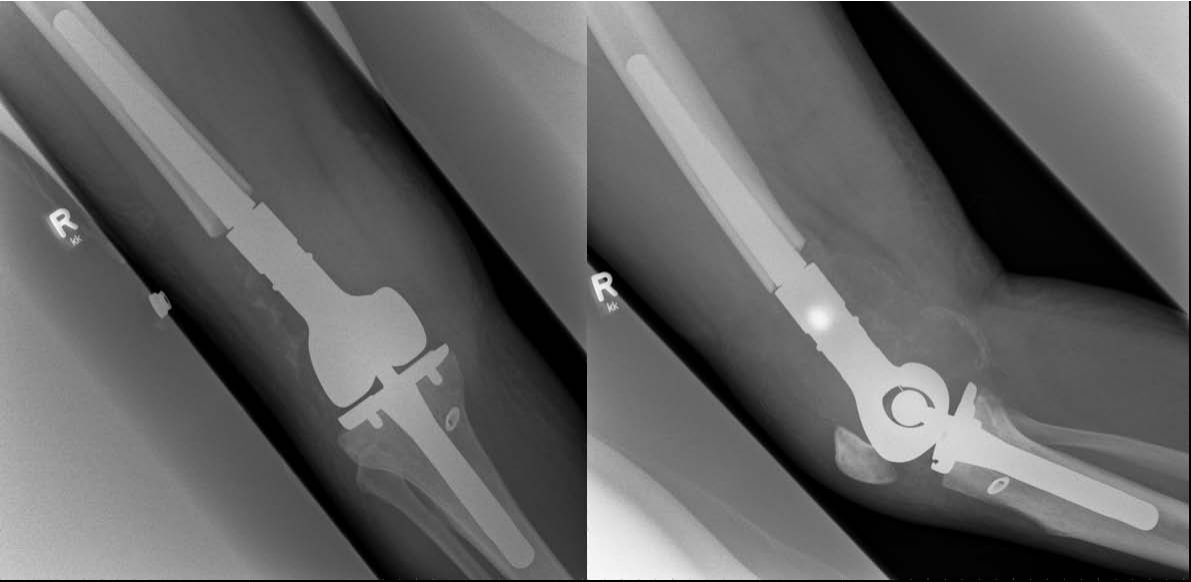
**Conventional X-ray imaging in anteroposterior (a) and lateral (b) projection after the implantation of a silver-coated non-cemented modular knee joint prosthesis (MUTARS)**.

**Figure 5:**
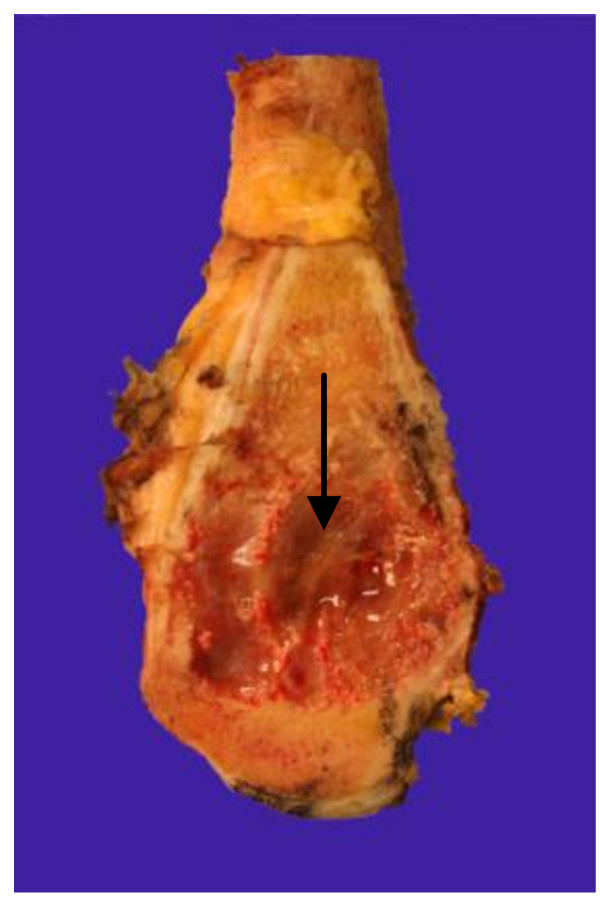
**Saggital section at the medial femoral condyle**. It shows the presence of an intraosseous tumor of up to 4.8 cm in size (black arrow) with brown, partially myxoid cut surface.

**Figure 6:**
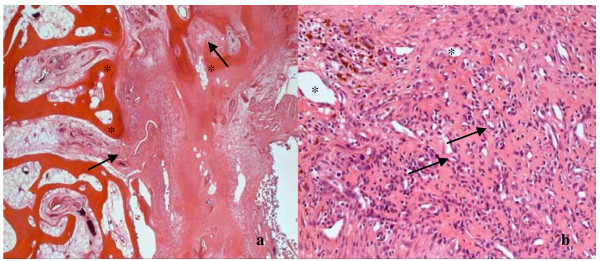
(a) Histology of the resected tumor still shows good vascularisation (*) and moderate regression of the tumor tissue with about 25% vital cells (black arrows) after neoadjuvant therapy; HE, 100-fold magnification. (b) Strong trabeculae (*) and collagen fibres (**Sharpey-like Fibers**; black arrows) that attached the tendon graft tightly to the bone; HE, 50-fold magnification.

An en bloc-resection of the distal femur 12 cm proximal to the knee joint including adjacent joint capsule and tumor-infiltrated soft tissue was performed. Resection was performed 1.5 cm below the tibial plateau. For subsequent distal femur reconstruction, a silver-coated non-cemented modular knee joint prosthesis (MUTARS, Implantcast, Buxtehude, Germany) was used (Figure. 4a and 4b). On the medial side, covering of soft-tissue defects with a flap was unnecessary. Postoperative mobilisation was conducted under partial weight bearing of 15 kg using 2 forearm crutches for the period of six weeks. The peri- and postsurgical course was regular and free of complications. Macroscopic pathological assessment showed a brown glassy tumor of up to 4.8 cm in size with a centre at the medial femoral condyle (Figure 5). The tumor reached the subchondral region. The distance from tumor to proximal bone resection margin was 6.5 cm, to proximal soft tissue resection margin 5.2 cm, to distal soft tissue resection margin 3 cm, to ventral soft tissue resection margin 2.5 cm and to dorsal soft tissue margin 3.5 cm. The tibial plateau was tumor-free. Histological examination of the specimen showed the proven malignant fibrous histiocytoma which had been completey resected. The site of semitendinosus tendon graft insertion showed a bone with strong trabeculae. Starting from the graft tunnel, collagen fibres (Sharpey-like Fibers) that attached the tendon graft tightly to the bone [11] could be demonstrated up to about 1.2 cm into the surrounding bone (Figure 6a). The moderately regressive MFH matching grade IV of Salzer-Kuntschik classification [12] indicated an only moderate response to neoadjuvant chemotherapy (Figure 6b). It was therefore decided by the interdisciplinary tumor conference together with the osteosarcoma register to carry on adjuvant chemotherapy according to the EURAMOS 1 protocol. 6 months after the implantation of the tumor-prosthesis the patient presented in our clinic. The postsurgical course was still free of complications and the patient was satisfied.

## Conclusions

Osseous malignant fibrous histiocytoma has been described for the first time in 1972 by Feldman and Norman [13]. Its defined pathohistological characteristics allow the distinction from other primary malignant bone tumors. Among the primary malignant bone tumors, MFH belongs to the rare ones with less than 5%. Since several decades, endoprostheses and orthopaedic implants have been used very successfully for treatment of degenerative diseases and traumatic injuries. The components of the used materials are considered as biologically inert.

In the literature, only 2 cases of malignant degeneration after ACL have been described so far. Sirveaux et al. [14] reported a 19-year-old male patient who underwent arthroscopic anterior cruciate ligament reconstruction using the patellar tendon in 1993. Tibial and femoral graft fixation was performed using a metal interference screw. 6 years after surgery, a pleomorphic malignant fibrous histiocytoma of the medial soft tissue was diagnosed at the operated knee joint. Because suture and metal particles were found in the sarcoma, the authors suspected a connection between the metal interference screws or the drilling of the graft tunnels, respectively, and the emergence of malignant fibrous histiocytoma. Caron et al. [15] reported a leiomyosarcoma at the distal femur 12 years after anterior cruciate ligament reconstruction. Also in this case a joint adjacent fixation with a metal interference screw had been performed. The sarcoma was located close to the interference screws. The authors consider it to be very unlikely that there is a connection between the malignant degeneration and the interference screw fixation, as the fixation material was not at the midpoint of the leiomyosarcoma. In the present case history, a fixation distant to the joint line with Endobutton and Suture Disc was used. The MFH was localised in the medial femoral condyle, far from the femoral drill tunnel. In the MFH, suture or metal particles could not be detected. A causal relationship between the Endobutton and the development of malignancy is most unlikely, as the Endobutton was positioned rather far from the MFH.

**Figure 7:**
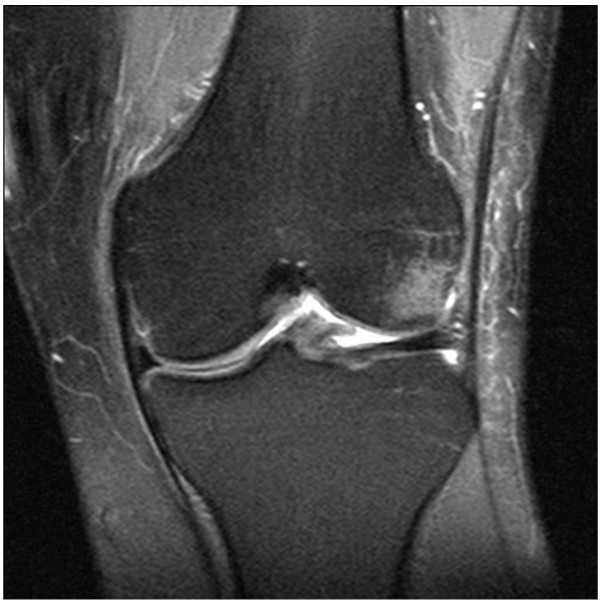
**Coronary MRI sequence**. Non-appearance of MFH before ACL rupture in 2006. Bone bruise in the lateral femoral condyle.

If a former trauma can favour MFH development remains unclear. Bader et al. [16] reported a 14-year-old girl who underwent plate osteosynthesis because of supracondylar femur fracture. 10 months after metal removal, a refracture and detection of an MFH at the same location occured. In a different case, Joss et al. [17] reported a 25-year-old patient with post-traumatic soft tissue MFH at the elbow after a severe elbow contusion. Both authors see a connection between the initial trauma and MFH development. In the present case history, the question cannot be clarified wether the MFH is a primary one or if there is an association with the initial trauma. However, regarding the latency period between initial trauma and appearance of pain, we consider a connection to be unlikely. By reanalysing the MR images taken in 2005, MFH presence prior to anterior cruciate ligament reconstruction could be excluded (Figure 7).

As the five-year survival rate is 10 to 30% if the MFH is treated only locally, neoadjuvant and adjuvant chemotherapy was performed analogous to the treatment of osteosarcoma in several studies [18]. The multi-modal therapy raised the five-year survival rate to 60%. Radiotherapy has only limited effectiveness in the treatment of highly malignant sarcomas and should only be applied if the tumor cannot be removed surgically. In the present case 2 cycles of chemotherapy were performed 10 weeks preoperatively, using adriamycin, doxorubicin, cisplatin and high dose-methotrexate with folic acid rescue. Under neoadjuvant chemotherapy, the MFH showed only moderate regressive changes (10 -- 50% vital tumor cells). The pain, however, had completely disappeared between neoadjuvant chemotherapy and surgical removal of the tumor.

## Abbreviations

MFH: malignant fibrous histiocytoma; MRI: magnetic resonance imaging; EURAMOS: The European and American Osteosarcome Study Group; MUTARS: Modular Universal Tumor And Revision System; Bq: Becquerel; Tc: Technetium; HDP: hydroxymethylene diphosphonate; HE: haematoxylin-eosin; ROM: range of motion; ACL: anterior cruciate ligament

## Competing interests

The authors declare that they have no competing interests.

## Authors' contributions

TE and JS were the major contributors in writing this manuscript. PR was the referent pathologist for this case. TJH diagnosed, investigated, followed up and managed the patient. TE, SFW and MDS performed the surgery on the patient. All authors read and approved the final manuscript.

## Consent

Written informed consent was obtained from the patient for publication of this case report and accompanying images. A copy of the written consent is available for review by the Editor-in-Chief of this journal.

## Pre-publication history

The pre-publication history for this paper can be accessed here:

http://www.biomedcentral.com/1471-2407/10/264/prepub
